# Pan‐cancer analysis identifies *TERT* alterations as predictive biomarkers for immune checkpoint inhibitors treatment

**DOI:** 10.1002/ctm2.109

**Published:** 2020-06-20

**Authors:** Tao Jiang, Qingzhu Jia, Wenfeng Fang, Shengxiang Ren, Xiaoxia Chen, Chunxia Su, Li Zhang, Caicun Zhou

**Affiliations:** ^1^ Department of Medical Oncology, Shanghai Pulmonary Hospital & Thoracic Cancer Institute Tongji University School of Medicine Shanghai China; ^2^ Department of Pulmonary Medicine, Shanghai Respiratory Research Institute, Zhongshan Hospital Fudan University Shanghai China; ^3^ Institute of Cancer, Xinqiao Hospital Third Military Medical University Chongqing China; ^4^ State Key Laboratory of Oncology in South China Collaborative Innovation Center for Cancer Medicine Department of Medical Oncology Sun Yat‐sen University Cancer Center Guangzhou China

**Keywords:** biomarker, immune checkpoint inhibitors, immune microenvironment, *TERT*

**Dear Editor,**

Immune checkpoint inhibitors (ICIs) targeting cytotoxic T lymphocyte antigen‐4 (CTLA‐4), or programmed cell death protein 1 (PD‐1) and its ligand (PD‐L1) interaction achieve a significant improvement in overall survival (OS) and revolutionize treatment paradigms in many types of cancers.^1^, ^2^ Despite of the durable antitumor effect, ICIs could only benefit a minority of patients (∼20%) without effective predictive biomarkers.^3^ Therefore, there is an urgent need to develop novel biomarkers for the majority of patients, who do not respond to ICIs monotherapy. Telomerase reverse transcriptase (*TERT*), as the catalytic subunit of telomerase, plays a critical role in modulating telomerase activity and immortalization of cancer cells.^4^, ^5^, ^6^, ^7^ It plays a crucial role in the tumorigenesis, cancer cell proliferation, invasion, and DNA damage response.^4^, ^7^ Recently, an elegant study found that short telomere length could cause a primary T cell immunodeficiency,^8^ suggesting the contribution of telomere length to T cell apoptosis and function. Therefore, it is valuable to investigate the predictive value of *TERT* alterations for ICIs treatment outcome in multiple cancers.

We identified a cohort of 43 910 cancer patients with 46 237 sequenced tumor samples, together with sequenced data and collected clinical information from the cBioPortal online database (https://www.cbioportal.org) (Figure S1). The prevalence of *TERT* alterations was 6.7%, with patients with thyroid cancer having the highest levels of *TERT* alterations (60.2%, 139/231; Figure S2). Most of the alterations were somatic mutations (73.1%, 2258/3091), especially promoter mutations (75.0%, 1693/2258). We then investigated the prevalence and spectrum of *TERT* alterations in early‐stage (TCGA cohort, N = 10 967) and advanced‐stage cancers (MSK‐IMPACT cohort, N = 10 945). The results showed that advanced‐stage cancers (15.2%, 1659/10 945) had significantly higher frequency of *TERT* alterations than early‐stage cancers (5.5%, 604/10 967; *P* < .0001). Most detected *TERT* alterations were amplifications in early‐stage cohort (Figure S3A), whereas most were *TERT* somatic mutations including promoter mutations in advanced‐stage cancers (Figure S3B). In MSK‐IMPACT cohort,^9^ we found that TMB of patients with *TERT* alterations was significantly higher than those without these alterations (17 vs 6 mut/Mb, *P* < .0001; Figure S4A). This was validated in the ICI‐treated cohort that included 1661 patients (TMB of *TERT* alterations vs wild type: 20 vs 9 mut/Mb, *P* < .0001; Figure S4B). Notably, cancers with multiple *TERT* alterations had the highest TMB level in both cohorts (Figures S4C and S4D).

Next, we evaluated the association between *TERT* alterations and clinical outcome. We first found that patients with *TERT* alterations showed a significantly shorter OS (38 vs 113 months; HR = 1.90; 95% CI, 1.73‐2.09; *P* < .0001; Figure 1A) than those without in whole group. In the ICI treatment cohort,^10^ we first identified 1661 patients with different cancers receiving ICI therapy and 521 of them with *TERT* alterations. Although clinicopathological features, including age, sex, sample type, and tumor purity, were not well balanced (Table S1), patients with *TERT* alterations had a substantially prolonged OS of 24 versus 17 months in the wild‐type group (HR = 0.78; 95% CI, 0.68‐0.91; *P* = .0016; Figure 1B). Although *TERT* alterations were associated with higher level of TMB, multivariate analysis revealed that *TERT* alterations were associated with markedly longer OS than wild type independent of TMB (HR = 0.77; 95% CI, 0.67‐0.91; *P* = .0020; Table S2). Notably, we also observed the association between *TERT* alterations and prolonged OS in patients with microsatellite‐stable solid tumors (not reached vs 20 months; HR = 0.35; 95% CI, 0.14‐0.88; *P* = .0248; Figure S5). Subgroup analysis revealed that patients with *TERT* promoter mutations also had the better OS than those with *TERT* wild type (22 vs 17 months, *P* = .0225; Figure 1C). Interestingly, in nonsmall‐cell lung cancer (NSCLC) treated with ICI, patients with *TERT* promoter mutations had the longest progression‐free survival (PFS) than other alterations and wild‐type groups (7.9 vs 2.6 vs 3.2 months; *P* = .0765; Figure 1D). To validate the abovementioned predictive value of *TERT* subtypes in NSCLC, we independently identified 158 patients with advanced NSCLC who received ICI monotherapy from three medical centers. Clinicopathological parameters were well balanced between *TERT* alterations and wild‐type groups (Figure 1E). Similarly, we found that patients with *TERT*‐altered NSCLC had significantly better objective response rate (35.7% vs 17.4%; *P* = .044) and prolonged PFS than wild type (8.8 vs 3.1 months; *P* = .0248; Figure 1F). Moreover, patients with *TERT* promoter mutations had the longest PFS than other alterations and wild‐type groups (9.7 vs 6.3 vs 3.1 months; *P* = .0333; Figure 1G).

**FIGURE 1:**
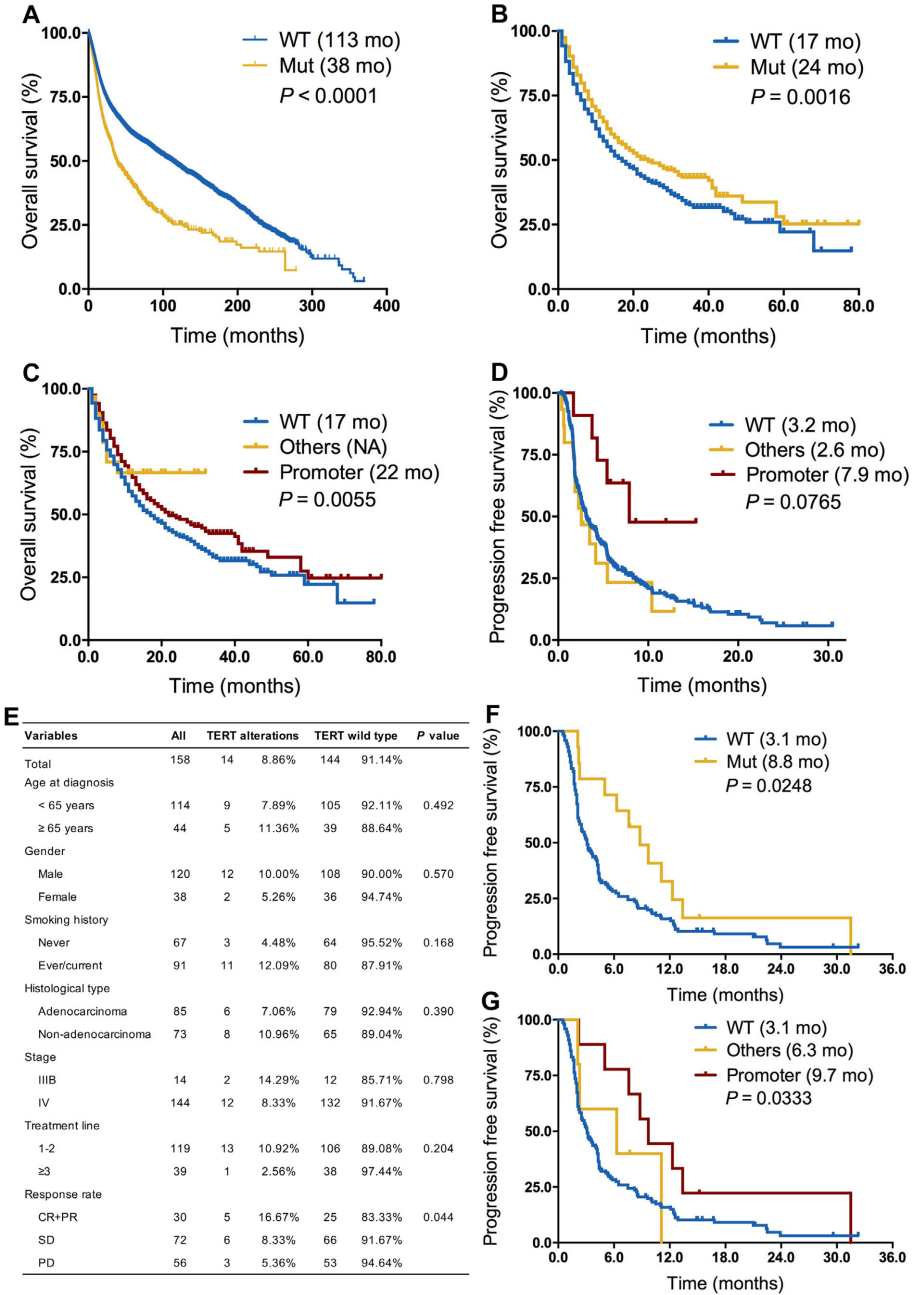
Association between *TERT* alterations and clinical outcome. A, Prognostic value of *TERT* alterations in all cancers. B, Predictive value of *TERT* alterations in patients who received ICI therapy. C, Subgroup analysis of the predictive value of *TERT* alterations subtypes in patients who received ICI treatment. D, Subgroup analysis of the predictive value of *TERT* alterations subtypes in nonsmall‐cell lung cancer patients who received ICI treatment. E, Baseline features of NSCLC patients with *TERT* alterations versus wild type from real‐world cohort. F, Predictive value of *TERT* alterations in patients with advanced NSCLC who received ICI monotherapy in real‐world cohort. G, Subgroup analysis of the predictive value of *TERT* alterations subtypes in patients with advanced NSCLC who received ICI monotherapy in real‐world cohort Abbreviations: CR, complete response; Mut, somatic mutations; PD, disease progression; PR, partial response; SD, stable disease; WT, wild type.

To depict the tumor immune microenvironment of *TERT*‐altered tumors, we surveyed the relationship between *TERT* alterations and eight common immune infiltrates including total CD3^+^ T cells, CD8^+^ T cells, cytotoxic lymphocytes, B lineage, NK cells, monocytic lineage, neutrophils, and myeloid dendritic cells across different cancer types (Figure 2). The results showed that tumor‐infiltrating CD8^+^ T cells, especially cytotoxic lymphocytes, were generally more abundant in the *TERT*‐altered tumors compared with those in the wild‐type tumors across multiple cancer types (Figure 2A‐D), whereas neutrophils were lower in *TERT*‐altered tumors than in wild type (Figures 2A and 2I). The results of signaling pathways in HALLMARK gene set collection showed that several signatures associated with antitumor immunity including DNA repair, unfolded protein response, E2F targets, cholesterol homeostasis, and so on were significantly higher in *TERT*‐altered tumors, whereas hallmarks associated with immune inhibitory function including hedgehog pathway, NOTCH signaling, transforming growth factor‐β (TGF‐β) signaling, angiogenesis, hypoxia, and so on were obviously higher in *TERT* wild‐type tumors (Figure 2J).

**FIGURE 2:**
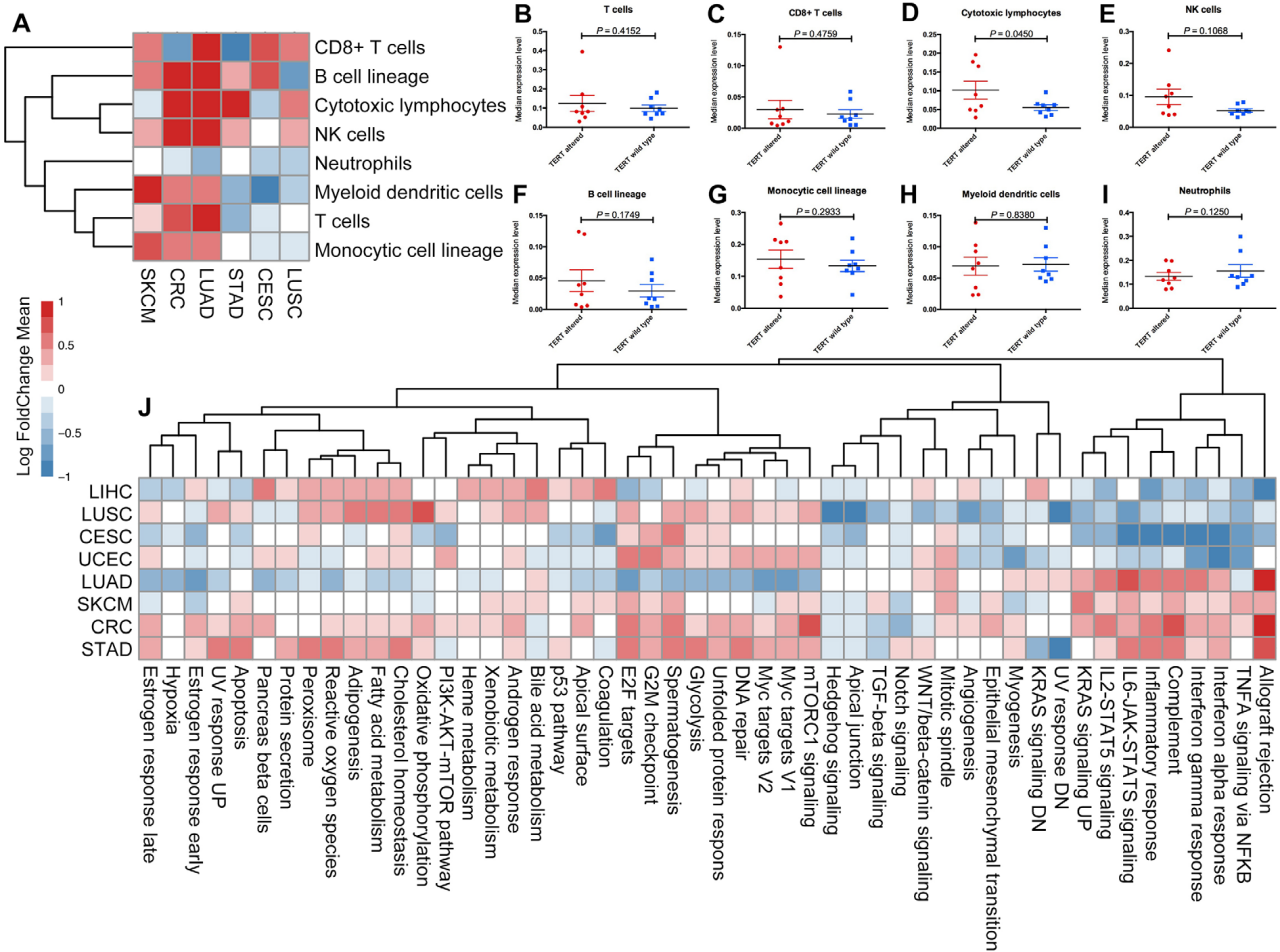
Immune landscape analysis of *TERT*‐altered and wild‐type tumors. A, Heatmap depicting the log_2_‐transformed fold change in the mean tumor‐infiltrating leukocytes MCP‐counter scores of the *TERT*‐altered tumors compared to *TERT* wild‐type tumors across different cancer types. B, Comparison of T cell abundance between the *TERT*‐altered and *TERT* wild‐type tumors. C, Comparison of CD8^+^ T cell abundance between the *TERT*‐altered and *TERT* wild‐type tumors. D, Comparison of cytotoxic lymphocyte abundance between the *TERT*‐altered and *TERT* wild‐type tumors. E, Comparison of NK cell abundance between the *TERT*‐altered and *TERT* wild‐type tumors. F, Comparison of B lineage abundance between the *TERT*‐altered and *TERT* wild‐type tumors. G, Comparison of monocytic lineage abundance between the *TERT*‐altered and *TERT* wild‐type tumors. H, Comparison of myeloid dendritic cell abundance between the *TERT*‐altered and *TERT* wild‐type tumors. I, Comparison of neutrophil abundance between the *TERT*‐altered and *TERT* wild‐type tumors. J, Heatmap depicting the mean differences in immune‐related hallmarks between the *TERT*‐altered and *TERT* wild‐type tumors across different cancer types Abbreviations: CESC, cervical squamous‐cell carcinoma and endocervical adenocarcinoma; CRC, colorectal cancer; LIHC, liver hepatocellular carcinoma; LUAD, lung adenocarcinoma; LUSC, lung squamous‐cell carcinoma; MCP, microenvironment cell populations; SKCM, skin cutaneous melanoma; STAD, stomach adenocarcinoma; UCEC, uterine corpus endometrial carcinoma.

In summary, the current study first provides the evidence that *TERT* alterations were associated with enhanced tumor immunogenicity and inflamed antitumor immunity, which result in prolonged OS in cancer patients treated with ICIs. The predictive value of *TERT* alterations was independent of tumor mutational burden and microsatellite status, suggesting that *TERT* alterations could be considered as a potential pan‐cancer predictive biomarker for ICI treatment. For the future, we still need to investigate the exact molecular mechanism, and large‐scale, prospective studies are also warranted.

## CONFLICT OF INTEREST

The authors declare no conflict of interest.

## Supporting information

Supporting Information
