# Supplementary material for: Pan‐cancer analysis identifies TERT alterations as predictive biomarkers for immune checkpoint inhibitors treatment
Source: Clin Transl Med. 2020 Jun 20;10(2):e109. doi: 10.1002/ctm2.109 (PMC7403829; doi:10.1002/ctm2.109)
Supplement: Supplementary file 1 — Supporting Information [file CTM2-10-e109-s001.pdf]

Supplemental Materials

Pan-cancer analysis identifies *TERT* alterations as predictive biomarkers for immune checkpoint inhibitors treatment

Tao Jiang, Qingzhu Jia, Wenfeng Fang, Shengxiang Ren, Xiaoxia Chen, Chunxia Su, Li Zhang, Caicun Zhou

Supplemental Figure S1……………………………………………………..2

Supplemental Figure S2……………………………………………………..3

Supplemental Figure S3……………………………………………………..4

Supplemental Figure S4……………………………………………………..5

Supplemental Figure S5……………………………………………………..6

Supplemental Table S1………………………………………………………7

Supplemental Table S2………………………………………………………8

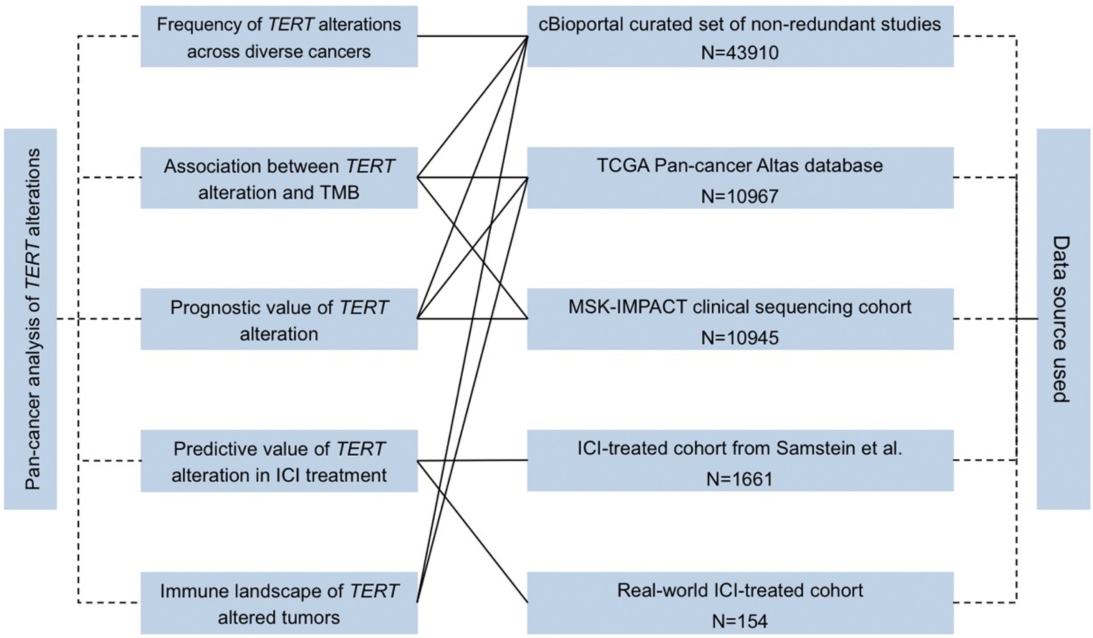

Supplemental Figure S1. Flowchart of the sequenced data and clinical cohort. The connected solid line between analysis aim (middle left) and data source (middle right) means the used cohort by this analysis.

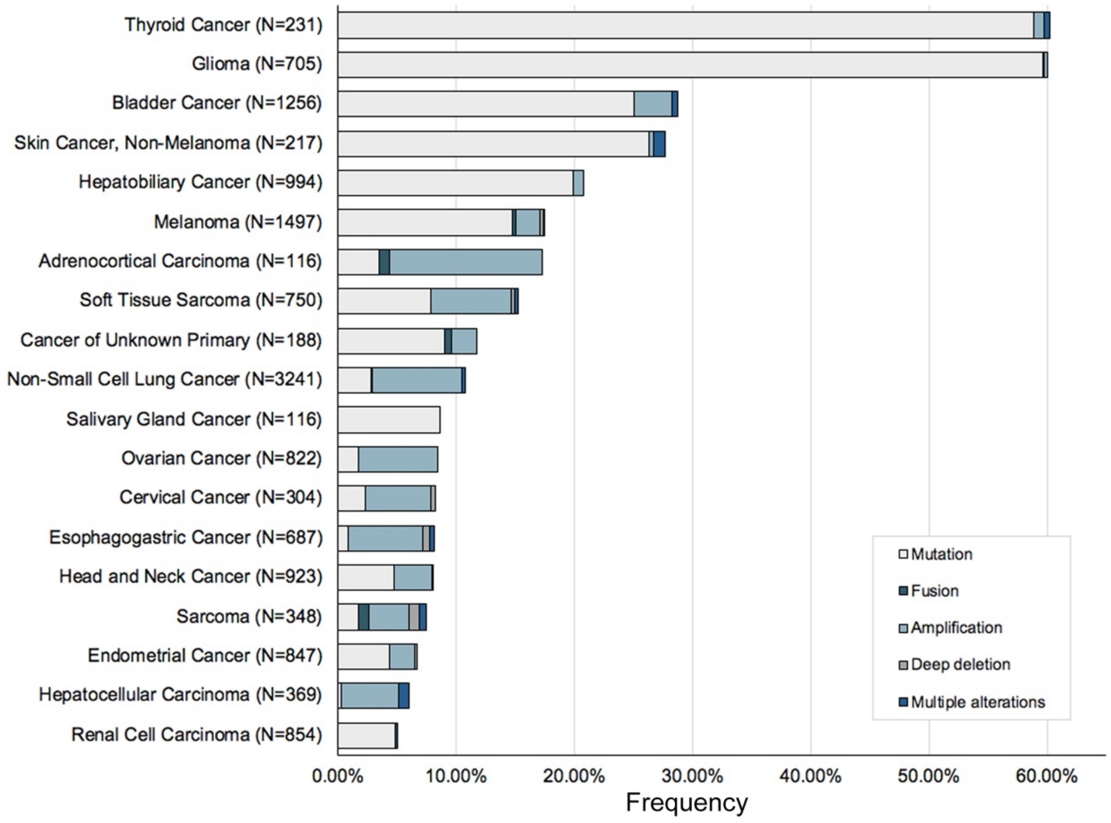

Supplemental Figure S2. Prevalence of *TERT* alterations in different cancers.

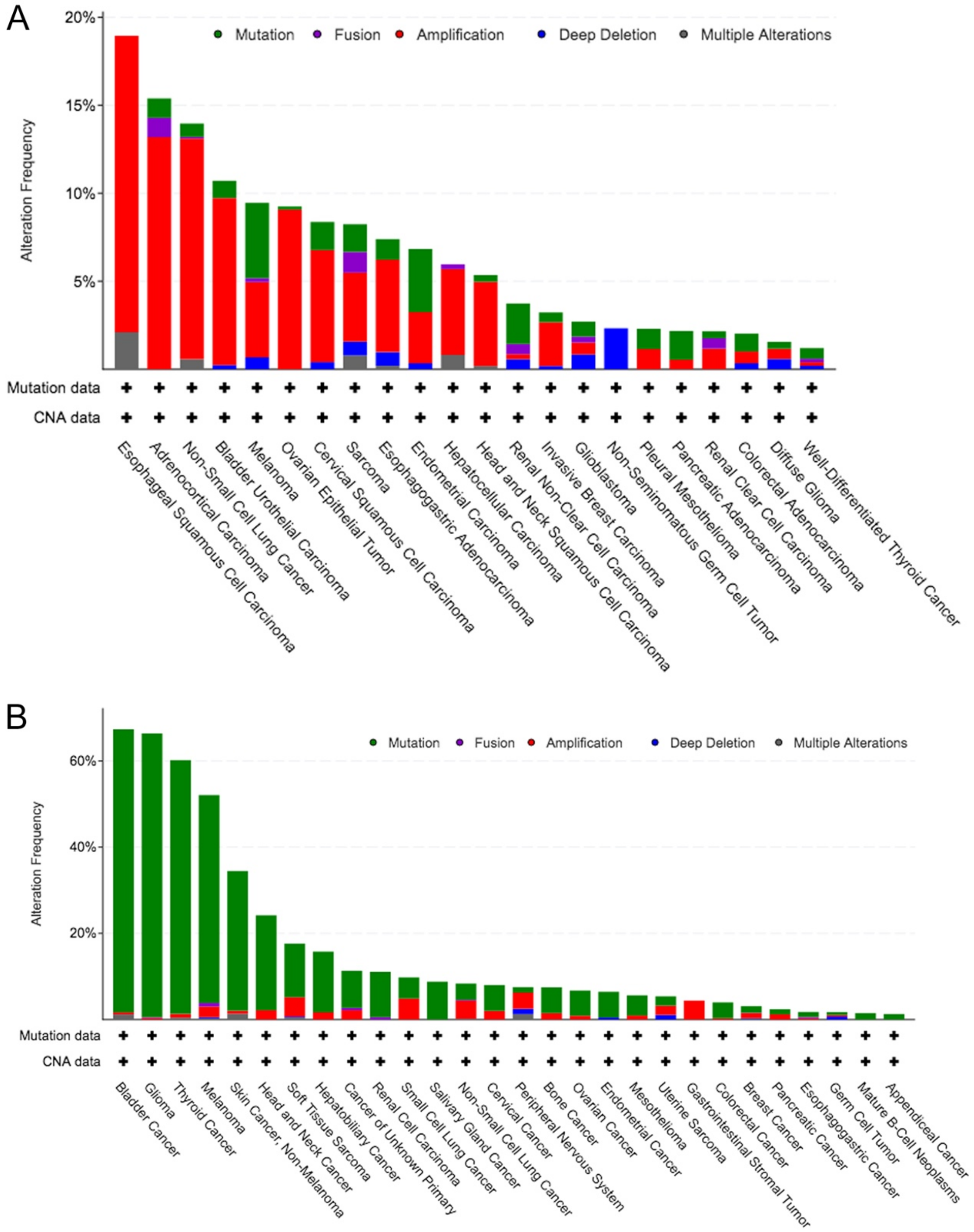

Supplemental Figure S3. The frequency of *TERT* alterations in early-stage cancer (A) and advanced-stage cancer (B).

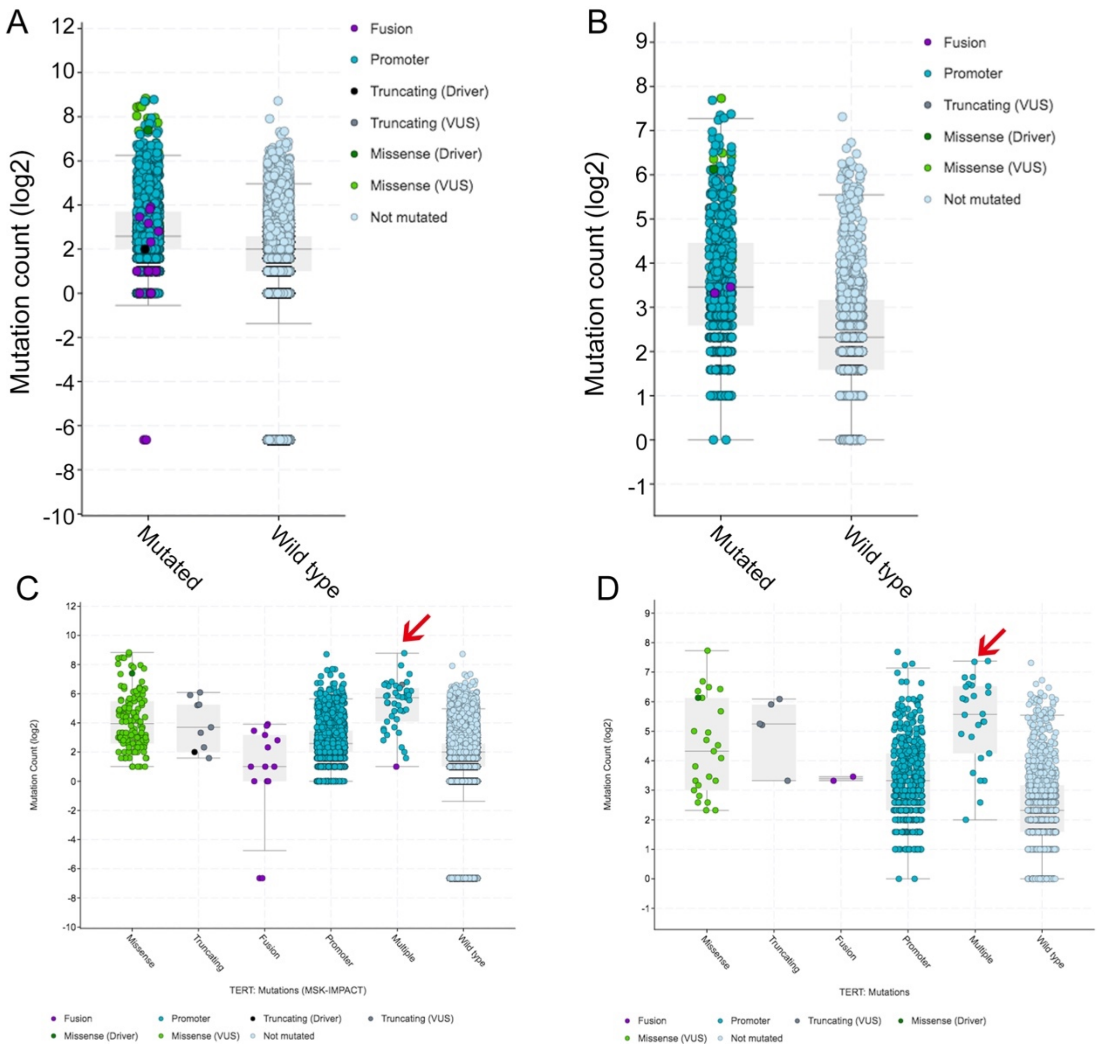

Supplemental Figure S4. Association between *TERT* alterations and tumor mutation burden (TMB) across diverse types of cancer. A. The association between TMB and *TERT* alterations in all cancers; B. The association between TMB and *TERT* alterations in immune checkpoint inhibitor (ICI) treated cohort. The association between TMB and *TERT* alterations subtypes in MSK-IMPACT cohort (C) and 1661 patients with advanced cancer received immune checkpoint inhibitors treatment cohort (D).

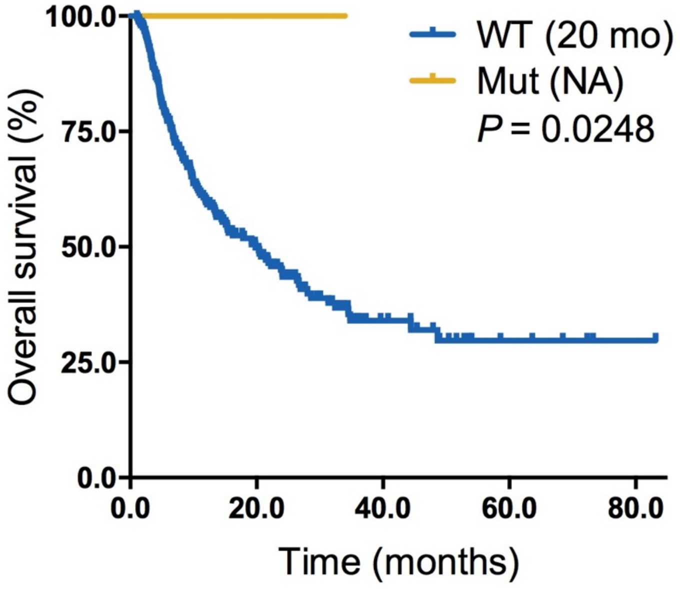

Supplemental Figure S5. Predictive value of TERT alterations in patients microsatellite-stable solid tumors received ICI treatment.

Supplemental Table S1. Baseline characteristics of the study population.

Variables	All cases	TERT alterations		TERT wild type		*P* value
Total	1661	521	31.37%	1140	68.63%	
Age at diagnosis						
< 65 years	922	266	28.85%	656	71.15%	0.014
≥ 65 years	738	255	34.55%	483	65.45%	
NA	1	0	0.00%	1	100.00%	
Gender						
Male	627	365	58.21%	262	41.79%	< 0.001
Female	1034	156	15.09%	878	84.91%	
Sample type						
Primary	930	238	25.59%	692	74.41%	< 0.001
Metastasis	731	283	38.71%	448	61.29%	
Drug type						
PD-1/PDL-1	1307	381	29.15%	926	70.85%	0.013
CTLA4	99	43	43.43%	56	56.57%	
Combo	255	97	38.04%	158	61.96%	
Tumor purity						
< 50	826	195	23.61%	631	76.39%	< 0.001
≥ 50	770	306	39.74%	464	60.26%	
NA	65	20	30.77%	45	69.23%	
Mutation count						
Median (range)	6 (1-212)	11 (1-212)		5 (1-159)		< 0.001
TMB score						
< 10	1173	257	21.91%	916	78.09%	< 0.001
≥ 10	488	264	54.10%	224	45.90%	

TMB, tumor mutation burden; NA, not applicable.

Supplemental Table S2. Multivariate analyses of clinical parameters on overall survival.

Factor	HR (log rank)	95% CI	*P* value
Sex (Female/male)	1.134	0.986-1.303	0.078
Age (<65/≥65)	0.996	0.868-1.142	0.951
Therapy (combo/mono)	0.561	0.453-0.695	<0.001
Tumor purity(≥50/<50)	0.854	0.745-0.979	0.024
TMB score (≥10/<10)	0.577	0.489-0.681	<0.001
Mutation count (≥6/<6)	0.820	0.716-0.940	0.004
TERT (alteration/wild type)	0.770	0.670-0.905	0.002

mono, monotherapy; combo, combination therapy; TMB, tumor mutation burden.
